# Supplementary material for: A set of multi-entry identification keys to African frugivorous flies (Diptera, Tephritidae)
Source: Zookeys. 2014 Jul 24;(428):97–108. doi: 10.3897/zookeys.428.7366 (PMC4143993; doi:10.3897/zookeys.428.7366)
Supplement: Supplementary material 6 — Key to Ceratitis [file zookeys-428-097-s006.zip › SF6_ZooKeys_key to Ceratitis/key/SF6_ZooKeys_key to Ceratitis/Media/Html/Ceratitis ealensis.htm]

***Ceratitis*** (***Ceratalaspis***)
***ealensis*** **De Meyer & Copeland**

*Ceratitis* (*Ceratalaspis*) *ealensis*
De Meyer & Copeland, 205 : 1293.

Body
length. 5.98 (5.15-6.55) mm; wing length. 5.75 (5.50-5.95) mm.

Female

Head.
Antenna yellow. First flagellomere 2 times as long as pedicel. Arista with
short rays, longest dorsal rays usually about as long as basal width of arista;
if somewhat longer never twice as long as basal width. Frons yellow; with short
scattered yellow setulae. Face yellowish white, below lower eye margin darker
yellow. Genal setulae and genal seta yellow to orange, well developed.

Thorax.
Postpronotal lobe yellow; without spot. Scutal pattern, ground colour yellow,
with distinct black markings but the latter strongly reduced; prescutellar markings
white, separate or merged; with yellow microtrichosity and pilosity. Scapular
setae yellow-orange. Scutellum yellow-white, basally without black spots,
apically with three separate black spots, extending to basal half. Subscutellum
yellow with three separate black spots. Anepisternum yellow; setulae yellow;
two black anepisternal setae, occasionally with a third, less developed and
dark orange, seta below two black ones.

Legs.
Yellow; setation yellow to orange. Fore femur with ventral setae orange.

Wing.
Bands mainly yellowish with some brown patches. Anterior apical and discal
bands not separate; subapical band joined to discal band; posterior apical band
absent; crossvein R-M at basal third of discal medial cell.�

Abdomen.
Yellow; pilosity mainly yellow. Tergites 2 and 4 with pale grey band occupying
posterior third to half of tergite. Tergite 5 with longer black setulae along
posterior margin. Oviscape about as long as preabdomen. Aculeus 8 times longer
than wide, apical part with two large rounded indentations, tip with small
depression.

Male

As
female.

(Description
after De Meyer & Copeland, 2005)
